# Hepatocyte activation and liver injury following cerebral ischemia promote HMGB1-mediated hepcidin upregulation in hepatocytes and regulation of systemic iron levels

**DOI:** 10.1038/s12276-024-01314-y

**Published:** 2024-10-01

**Authors:** Dashdulam Davaanyam, Song-I Seol, Sang-A Oh, Hahnbi Lee, Ja-Kyeong Lee

**Affiliations:** Department of Anatomy, Inha University School of Medicine, Incheon, 22212 Korea

**Keywords:** Diseases of the nervous system, Stroke

## Abstract

We previously reported that high mobility group box 1 (HMGB1), a danger-associated molecular pattern (DAMP), increases intracellular iron levels in the postischemic brain by upregulating hepcidin, a key regulator of iron homeostasis, triggering ferroptosis. Since hepatocytes are the primary cells that produce hepcidin and control systemic iron levels, we investigated whether cerebral ischemia induces hepcidin upregulation in hepatocytes. Following middle cerebral artery occlusion (MCAO) in a rodent model, significant liver injury was observed. This injury was evidenced by significantly elevated Eckhoff’s scores and increased serum levels of alanine aminotransferase (ALT) and aspartate aminotransferase (AST). Additionally, total iron levels were significantly elevated in the liver, with intracellular iron accumulation detected in hepatocytes. Hepcidin expression in the liver, which is primarily localized in hepatocytes, increased significantly starting at 3 h after MCAO and continued to increase rapidly, reaching a peak at 24 h. Interestingly, HMGB1 levels in the liver were also significantly elevated after MCAO, with the disulfide form of HMGB1 being the major subtype. In vitro experiments using AML12 hepatocytes showed that recombinant disulfide HMGB1 significantly upregulated hepcidin expression in a Toll-like receptor 4 (TLR4)- and RAGE-dependent manner. Furthermore, treatment with a ROS scavenger and a peptide HMGB1 antagonist revealed that both ROS generation and HMGB1 induction contributed to hepatocyte activation and liver damage following MCAO–reperfusion. In conclusion, this study revealed that cerebral ischemia triggers hepatocyte activation and liver injury. HMGB1 potently induces hepcidin not only in the brain but also in the liver, thereby influencing systemic iron homeostasis following ischemic stroke.

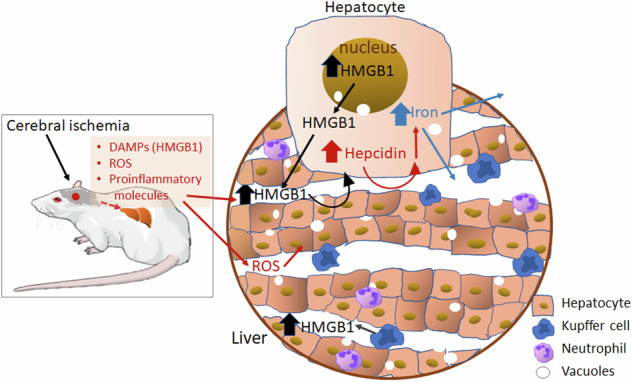

## HMGB1: A critical mediator of liver injury after ischemic stroke

Iron is vital for many body functions, but its quantity needs careful monitoring to avoid harm. The processes controlling iron, particularly after brain injuries like strokes, are not entirely known. Researchers studied how strokes affect liver function and iron control. They experimented on rats, causing strokes and then observing the impact on liver damage, iron quantities, and the production of hepcidin, a hormone crucial for iron control. This research used animal models to better comprehend these biological processes. The findings reveal that strokes can harm the liver and disrupt the body’s iron control by increasing hepcidin quantities. The researchers conclude that understanding these processes could help create treatments to manage iron quantities after a stroke, potentially improving stroke patients. This summary was initially drafted using artificial intelligence, then revised and fact-checked by the author.

## Introduction

Iron, the most abundant metallic element found in the human body, is distributed throughout various tissues and organs. Its involvement in fundamental biological processes, including mitochondrial respiration, cellular metabolism, oxygen transportation, DNA synthesis, neurotransmitter production, and myelin synthesis^1–4^, necessitates tight regulation of both intracellular and extracellular iron levels. Increased intracellular iron levels have been associated with various central nervous system (CNS) pathologies, including Alzheimer’s disease^5,6^, Parkinson’s disease^7,8^, and Huntington’s disease^9^. Neuronal damage arising from the dysregulation of cellular iron homeostasis and elevated iron levels has also been observed in animal models of stroke^10–12^ and stroke patients^13,14^.

In mammalian tissues, cellular iron homeostasis is maintained by a network of molecules involved in iron uptake into cells, iron storage and regulation within cells, and iron export from cells. Hepcidin, a key player in this regulatory system, is a peptide hormone that governs intracellular iron export by triggering the internalization and degradation of ferroportin (FPN), the only known iron exporter^15^. By inhibiting FPN, hepcidin effectively increases intracellular iron levels within target cells and restricts the release of iron into the bloodstream. Hepcidin is predominantly expressed in hepatocytes, macrophages, and duodenal enterocytes, which are cell types that play crucial roles in iron recycling, absorption, and storage^16^. Hepcidin expression is modulated by various factors, including changes in systemic iron levels, inflammation, erythropoiesis, hypoxia, and endoplasmic reticulum stress^16–19^.

In our previous study, we observed a pronounced and rapid increase in iron levels within the cortex of the ischemic hemisphere, accompanied by the upregulation of hepcidin expression in the brain within 3 h following middle cerebral artery occlusion (MCAO, 60 min)^20^. High mobility group box 1 (HMGB1), a prototypic danger-associated molecular pattern (DAMP), mediates local (brain) hepcidin upregulation and the subsequent iron surge, ultimately leading to ferroptosis in the postischemic brain^20^. In the same study, we also detected a significant accumulation of hepcidin in the serum 6 h after MCAO, which persisted for 7 days^20^. Given that HMGB1 accumulates in the brain parenchyma, cerebrospinal fluid (CSF), and serum following transient MCAO and plays a crucial role in exacerbating damage through proinflammatory effects^21–23^ HMGB1 is suggested to play a critical role in systemic hepcidin induction and the systemic regulation of iron levels after MCAO. A prolonged increase in serum hepcidin levels has been reported in various neurological conditions, including cerebral ischemia^13,24,25^ and intracerebral hemorrhage (ICH)^26^, potentially contributing to iron overload in the brain and other peripheral organs. However, the underlying mechanisms governing systemic hepcidin regulation and iron levels following cerebral ischemic injury remain to be elucidated.

Hepatocytes are the primary cell type responsible for the synthesis and release of hepcidin in response to elevated iron levels and inflammation within the body^16^. This study aimed to investigate whether liver injury occurs following cerebral ischemia (MCAO) and to determine whether hepatocytes upregulate hepcidin expression in response to cerebral ischemia. Additionally, we explored whether HMGB1 expression is induced in the liver after MCAO and plays a role in hepcidin upregulation in hepatocytes, ultimately contributing to the regulation of systemic iron levels.

## Materials and Methods

### Surgical procedure used for MCAO

Male Sprague‒Dawley rats (7-8 weeks old) were housed under diurnal lighting conditions and allowed food and tap water *ad libitum*. All animal studies were performed in strict accordance with the recommendations of the Guide for the Care and Use of Laboratory Animals published by the National Institutes of Health (NIH, USA, 2013) and the ARRIVE guidelines [http://www.nc3rs.org/ARRIVE (accessed on August 31, 2021)]. The animal protocol used was reviewed for ethical approval and approved by the INHA University Institutional Animal Care and Use Committee (INHA-IACUC) (approval number INHA180105-531-2). MCAO was induced as previously described^20^. Briefly, anesthesia was induced in 8-week-old male Sprague‒Dawley rats (250–300 g) using 5% isoflurane in 30% oxygen/70% nitrous oxide and maintained using 0.5% isoflurane in the same gas mixture during surgery. The right middle carotid artery was occluded for 1 h by advancing a nylon suture (4-0; AILEE, Busan, Korea) with a heat-induced bulb at its tip (approximately 0.3 mm diameter) along the internal carotid artery 20-22 mm from its bifurcation with the external carotid artery. This step was followed by reperfusion for up to 7 days. A thermoregulated heating pad and a heating lamp were used to maintain a rectal temperature of 37.0 ± 0.5 °C during surgery. After 12 h of reperfusion, the left femoral arteries were cannulated to obtain blood samples, which were analyzed for pH, PaO_2_, PaCO_2_, and the blood glucose concentration (I-STAT; Sensor Devices, Waukesha, WI, USA) (Table 1). Animals were randomly allocated to the following groups: sham (*n* = 17), MCAO 1 h (*n* = 4), MCAO 2 h (*n* = 4), MCAO 3 h (*n* = 14), MCAO 6 h (*n* = 10), MCAO 12 h (n = 20), MCAO 24 h (*n* = 14), MCAO 48 h (*n* = 10), MCAO 72 h (*n* = 10), MCAO 96 h (*n* = 7), MCAO + NAC, 3 h (*n* = 4), and MCAO + HMGB1 A box, 3 h (*n* = 4). Animals in the sham group underwent the same procedure, but their middle cerebral arteries were not occluded.

**Table 1:** Physiological parameter.

	Base (*n* = 3)	MCAO 12 h (*n* = 3)
pH	7.5 ± 0.1	7.6 ± 0.1
pO_2,_ mmHg	122.2 ± 10.6	132.7 ± 7.9
pCO_2,_ mmHg	33.1 ± 1.9	32.5 ± 1.7
Glucose, mg/dl	106.1 ± 3.3	99.2 ± 3.2
Rectal temperature, ° C	36.6 ± 0.2	36.4 ± 0.4

Values are means ± SD (*n* = 3). 1-way analysis of variance revealed no significant difference for any variance

### Drug administration

For the HMGB1 A box (HM-012, 5 μg/kg; HMGbiotech, Milano, Italy), the rats were anesthetized via an intramuscular injection of a mixture of ketamine (3.75 mg/100 g body weight) and xylazine hydrochloride (0.5 mg/100 g per body weight). A nose drop containing an HMGB1 A box (5 μg/kg) was carefully placed in each nostril of anesthetized animals (supine at a 90° angle) using a preautoclaved pipet tip (T-200-Y; Axygen, Union, CA, USA). The procedure was repeated until all dosages were administered, with 2-min intervals between applications. For N-acetyl cysteine (NAC; A7250, Sigma‒Aldrich, St. Louis, MO, USA), 150 mg/kg NAC was injected intraperitoneally.

### Staining with 2,3,5-triphenyl tetrazolium chloride (TTC)

Rats were sacrificed 3, 6, 12, 24, 48, 72, and 96 h after MCAO, and whole brains were dissected coronally into 2-mm slices using a metallic brain matrix (RBM-40000, ASI, Springville, UT, USA). The slices were immediately incubated in saline containing 2,3,5-triphenyl tetrazolium chloride (TTC, 2%) for 15 min at 37 °C and then stored in 4% paraformaldehyde (PFA, FUJIFILM Wako Pure Chemical, Osaka, Japan). The areas of infarcted tissue at 24 h after MCAO were measured using the Scion Image program (Informer Technologies Inc., Frederick, MD, USA).

### Cell culture and treatment

Alpha mouse liver 12 (AML12) cells, a mouse hepatocyte cell line, were grown in Dulbecco’s modified Eagle’s medium/F-12 (DMEM; Welgene, Daegu, Korea) supplemented with 1% penicillin and streptomycin (Gibco, Carlsbad, CA, USA), 1% insulin–transferrin–selenium (ITS; Gibco), 10% fetal bovine serum (FBS; HyClone, Logan, UT, USA) and dexamethasone (40 ng/ml, Sigma‒Aldrich) at 37 °C in a 95% air/5% CO_2_ humidified atmosphere. Cells were seeded in 6-well tissue culture plates (Corning, Corning, NY, USA) at a density of 2 × 10^4^ cells/well. Disulfide HMGB1 (dsHMGB1, HM-120, 100 ng/ml) and reduced HMGB1 (reHMGB1, HM-115, 100 ng/ml) were purchased from HMGBiotech Srl (Milano, Italy). The Toll-like receptor 4 (TLR4) antagonist (TLR4-IN-C34, 10 µM), C-X-C chemokine receptor type 4 (CXCR4) receptor antagonist (AMD3100, 5 µg/ml), and receptor for advanced glycation end products (RAGE) antagonist (FPS-ZM1, 500 nM) were purchased from Sigma‒Aldrich. Recombinant interlukin-6 (rIL-6, R&D Systems Inc., Minneapolis, MN, USA) was used as a positive control.

### Iron measurement

The total iron content in the liver tissues was determined using an Iron Assay Kit (Sigma‒Aldrich) according to the manufacturer’s instructions. Briefly, liver tissues were collected after 3, 6, 12, 24, 48, 72, and 96 h of reperfusion following MCAO (60 min). The collected tissues were homogenized in a 5× volume of iron assay buffer on ice and centrifuged (13,000 × *g*, 10 min) at 4 °C. The supernatant was collected, 5 µl of iron reducer was added to each sample, and the sample was incubated for 30 min at 37 °C. An iron probe (100 µl) was added to each sample and incubated for 60 min at 37 °C in the absence of light. The absorbance was measured at 593 nm using a microplate reader (Thermo Fisher Scientific, Waltham, MA, USA).

### Perls’ Prussian blue staining

Liver sections (30 μm thick) were fixed with 4% PFA, incubated with a 1:1 mixture of 1% potassium ferrocyanide and 2% hydrochloric acid, and counterstained using a 0.1% nuclear fast red solution (Abcam, Cambridge, MA, USA). Prussian blue-positive cells were observed using an optical microscope (Olympus IX83, 40x; Olympus Corporation, Tokyo, Japan).

### Reverse transcription‑quantitative polymerase chain reaction (RT‑qPCR)

Total RNA was extracted from rat liver tissues at 3, 6, 12, 24, 48, 72, and 96 h after MCAO using TRIzol reagent (Invitrogen, Life Technologies, Carlsbad, CA, USA). cDNA was synthesized using an iScript cDNA synthesis kit (Bio-Rad, Hercules, CA, USA) according to the manufacturer’s instructions. Target gene mRNA levels were determined by real-time PCR using TOPreal qPCR 2X PreMIX SYBR Green with low ROX (carboxyrhodamine) (Enzynomics, Daejeon, Korea). The PCR conditions used were as follows: 15 min at 95 °C, followed by 55 cycles of activation for 10 s at 95 °C and annealing/extension for 15 s at 55 °C. The primers used are listed in Table 2. PCR was performed in triplicate, and the threshold cycle numbers were averaged for each sample. The cycle threshold (Ct) values were normalized to those of GAPDH, and the Livak (2-ΔΔCt) method was used to calculate changes in target gene expression.

**Table 2:** Oligonucleotide primers used for qPCR analysis.

Gene (GenBank No.)	Oligonucleotide primer sequences	PCR product size (bp)
HAMP	5’-TCTCCTGCTTCTCCTCCTTG-3’	167
(NM_032541.2)	5’-AGATGCAGATGGGGAAGTTG-3’	
GAPDH	5’-AGATGCAGATGGGGAAGTTG-3’	363
(NM_001411843.1)	5’-AGATGCAGATGGGGAAGTTG-3’	

### Enzyme-linked immunosorbent assay (ELISA)

The levels of hepcidin and IL-6 in liver tissue and alanine aminotransferase (ALT) and aspartate aminotransferase (AST) in serum were assessed using ELISA kits (Cusabio, Houston, TX, USA) according to the manufacturer’s instructions. Tissue homogenates obtained from the liver were rinsed with phosphate-buffered saline (PBS), homogenized in 1 ml (for 100 mg tissue) of PBS, and centrifuged for 5 min at 5,000 rpm at −4 °C. The supernatants were immediately transferred to clean polypropylene tubes, and concentrations were determined using ELISA kits in accordance with the manufacturer’s instructions.

### Evaluation of malondialdehyde (MDA) concentrations

The MDA concentrations in cell lysates and in serum were assessed using the Lipid Peroxidation (MDA) Assay Kit (Sigma‒Aldrich, Cat #: MAK085) according to the manufacturer’s instructions. Briefly, MDA in the brain tissue and serum was reacted with thiobarbituric acid (TBA) to generate the MDA-TBA adduct; the MDA-TBA adduct was quantified fluorometrically (excitation/emission = 532/553 nm) on a microplate reader.

### Immunoblot analysis

Liver tissue homogenates or whole-cell lysates were extracted using radioimmunoprecipitation assay buffer (RIPA buffer) [50 mM Tris-HCl (pH 7.4), 150 mM NaCl, 1 mM EDTA, 0.5% NP40, 0.25% sodium deoxycholate, 0.5% Triton X-100, 10% glycerol, and Complete Mini Protease Inhibitor Cocktail tablet (Roche Diagnostics, Basel, Switzerland)]. Cell or tissue extracts were then loaded on 10~12% SDS‒PAGE gels and immunoblotted using the following primary antibodies: anti-FPN (1:1000, Abcam), anti-divalent metal transporter (DMT1, 1:3000, Biorbyt, Cambridge, MA, UK), anti-ferritin heavy chain (Ft-H, 1:2000, Santa Cruz Biotechnology, Dallas, TX, USA), anti-ferritin light chain (Ft-L, 1:2000, Santa Cruz Biotechnology), anti-HMGB1 (1:3000, Abcam), anti-α-tubulin (1:5000, Santa Cruz Biotechnology), anti-GAPDH (1:5000, Cell Signaling Technology, Danvers, MA, USA), and anti-β-actin (1:3000, Santa Cruz Biotechnology). Blots were detected using horseradish peroxidase (HRP)-conjugated anti-rabbit or anti-mouse secondary antibodies (1:2000, Merck Millipore, Burlington, MA, USA) and a chemiluminescence kit (Merck Millipore).

### Immunofluorescence and immunohistochemical staining

The liver tissue blocks were fixed in a 4% PFA solution for 2 days at 4 °C and postfixed in a 30% sucrose solution at 4 °C. Sections with a thickness of 30 μm were obtained using a vibratome, and immunological staining was performed. The sections were blocked with 5% FBS, 5% horse serum, and 2% albumin in 0.1% Triton X-100 for 1 h at room temperature. The following primary antibodies were diluted to 1:200 and incubated with the sections: mouse anti-HMGB1 (Santa Cruz Biotechnology), rabbit anti-CD68 (Biorbyt), mouse anti-CD68 (Abcam), and rabbit anti-hepcidin (Bioss Antibodies). After the incubation with the primary antibodies, the liver sections were washed with PBS and incubated with rhodamine-labeled anti-mouse IgG (1:300, Merck Millipore Corporation) for anti-HMGB1 and anti-CD68 and FITC-labeled anti-rabbit IgG (Thermo Fisher Scientific) for anti-hepcidin and anti-CD68 in PBS for 1 h at room temperature. Sections were mounted on slides using VECTASHIELD Antifade Mounting Solution containing 4′,6-diamidino-2-phenylindole (DAPI, Vector Laboratories, Burlingame, CA, USA) and examined under a Zeiss LSM 510 META microscope (Carl Zeiss Meditec AG, Jena, Germany). For immunohistochemical staining, paraffin-embedded liver sections were incubated with a rabbit anti-HMGB1 antibody (Novus Biologicals, Littleton, CO, USA) and anti-hepcidin antibody (Bioss antibodies), followed by an incubation with an HRP-labeled secondary antibody for 1 h at room temperature. Images were captured using a microscope (Olympus IX83).

### Morphometric assessment of liver damage/hematoxylin and eosin (H&E) staining

Liver tissues were fixed with 4% PFA, embedded in paraffin, and cut into 5 µm sections using a microtome. Deparaffinized sections were stained with H&E and observed under a light microscope (Olympus IX83, 40x). H&E-stained sections were evaluated blindly according to Eckhoff’s scoring system. Liver tissues were evaluated at 200x magnification for the severity of hepatic injury using an ordinal scale as follows: grade 0, minimal or no evidence of injury; grade 1, mild injury consisting of cytoplasmic vacuolation and focal nuclear pyknosis; grade 2, moderate to severe injury with extensive nuclear pyknosis, cytoplasmic hypereosinophilia, loss of intercellular borders, and mild-to-moderate neutrophil infiltration; and grade 3, severe injury with disintegration of hepatic cords, hemorrhage, and severe neutrophil infiltration.

### Statistical analysis

Two-sample comparisons were performed using Student’s *t-*test. Multiple comparisons were performed using one-way or two-way analysis of variance, followed by Tukey’s post hoc test. PRISM software 5.0 (Graph Pad Software Inc., San Diego, CA, USA) was used for all analyses, and the results are presented as the means ± SEMs. *p* values < 0.05 were considered to indicate statistical significance.

## Results

### Cerebral ischemia-induced liver damage

**Fig. 1:**
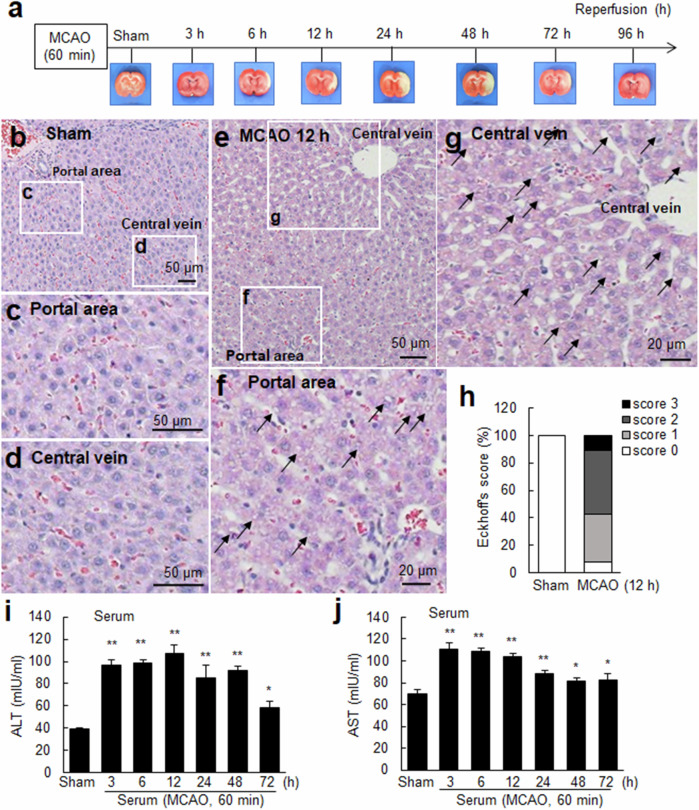
Liver damage after a cerebral ischemic insult (MCAO). **a** Schematic illustration of TTC staining depicting infarct formation in the postischemic rat brain at 3, 6, 12, 24, 48, 72, and 96 h of reperfusion following MCAO (60 min). Liver tissue samples were obtained from the sham control (**b**–**d**) and MCAO groups (**e**–**g**) at 12 h after MCAO, and H&E staining was performed. High-magnification images around the portal area and central vein, indicated by the white boxes in **b** (sham controls) and **e** (MCAO), are presented in **c**, **d**, **f**, and **g**. **h** The severity of liver damage was assessed using Eckhoff’s scoring system. Representative images are shown in **b**–**g**, and scores from Eckhoff’s scoring system are presented in h (*n* = 20 for the sham control and *n* = 64 for MCAO from 4 animals). The arrows indicate vacuoles in the hepatocytes. The scale bars in **b**–**e** represent 50 µm, and those in **f** and **g** represent 20 µm. Serum levels of ALT (**i**) and AST (**j**) were measured at 3, 6, 12, 24, 48, and 72 h after MCAO using ELISAs. The quantified results are presented as the means ± SEMs (*n* = 4). *p < 0.05 and **p < 0.01 compared with sham controls.

We investigated whether harmful changes occur in the liver following cerebral ischemia and whether hepcidin expression is induced in the liver using an animal model of MCAO (60 min) (Fig. 1a). The infarct volume measured at 12 and 24 h post-MCAO using TTC staining (Supplementary Fig. 1a, b), the mean modified neurological severity scores (mNSSs) (Supplementary Fig. 1c), and physiological parameters (Table 1) indicated that the MCAO animal model was successfully established. The histological examination with H&E staining revealed no signs of liver tissue damage in sham-operated animals (Fig. 1b-d). In contrast, vacuolization of hepatocytes was evident in the liver 12 h after MCAO (arrows, Fig. 1e–g). Additionally, hepatic cord destruction, sinusoidal congestion, and sinusoidal and central vein dilation were also observed in the livers of the MCAO group (Fig. 1e–g). Furthermore, inflammatory cell infiltration, particularly neutrophil infiltration, was observed in the liver parenchyma (Supplementary Fig. 2). At 12 h after reperfusion, severe liver tissue damage was observed, which was indicated by changes in Eckhoff’s scores (Fig. 1h). Importantly, at 3 h after MCAO, the level of ALT, a serum marker of liver function, was significantly higher in the MCAO group than in the sham control group (Fig. 1i). The elevated ALT levels persisted until 72 h after MCAO (Fig. 1i). Similarly, serum AST levels were also significantly increased in the MCAO group compared to the sham control group (Fig. 1j). ALT and AST levels also increased significantly in the liver tissue after MCAO, but these levels gradually decreased (Supplementary Fig. 3). Taken together, these results suggest that cerebral ischemic injury induces liver damage.

### Increased intracellular iron levels in the liver after cerebral ischemia

**Fig. 2:**
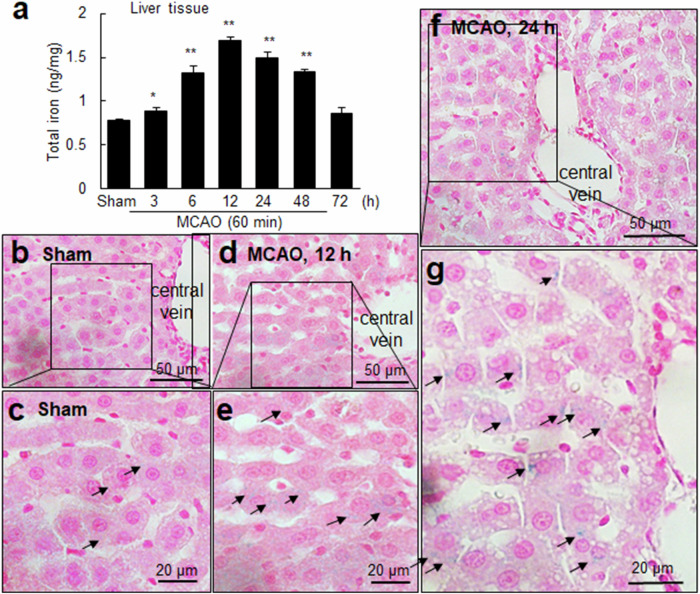
Total iron levels and intracellular iron localization in the liver tissue after MCAO. **a** Liver tissue samples were obtained 3, 6, 12, 24, 48, and 72 h after MCAO. The total iron content was measured using an iron assay kit. The results are presented as the means ± SEMs (*n* = 4). Liver tissue sections were prepared from the sham control (**b**, **c**) and MCAO groups (**d**–**g**) at 12 and 24 h after MCAO and stained with Perls’ Prussian blue and nuclear fast red (a nuclear counterstain). Prussian blue-positive cells are indicated by arrows. Representative images from three MCAO groups are shown. The scale bars in **b**, **d**, and **f** represent 50 µm, and those in **c**, **e**, and **g** represent 20 µm. *p < 0.05 and **p < 0.01 compared with the sham-operated control group.

Elevated serum iron levels have been reported in patients with ischemic stroke^24,25^ or ICH^26^; however, changes in iron levels in the liver after cerebral ischemia have not been reported. We used an iron assay kit to investigate whether total iron levels in the liver changed after MCAO. Iron levels in the liver tissue increased significantly as early as 3 h after MCAO and peaked at 12 h after MCAO (Fig. 2a). These elevated iron levels persisted for 48 h and returned to the basal level at 72 h after MCAO (Fig. 2a). When Prussian blue staining was performed to visualize intracellular iron in the liver tissue, barely detectable iron staining was observed in the sham control group (Fig. 2b, c). In contrast, intracellular iron localization was detected in hepatocytes at 12 h after MCAO and increased further at 24 h after MCAO (Fig. 2d–g). However, minimal iron staining was observed in Kupffer cells. Collectively, these findings indicate that an increase in the intracellular iron level occurs in the liver following cerebral ischemic injury.

### Alterations in iron-related protein expression in the liver after cerebral ischemia

**Fig. 3:**
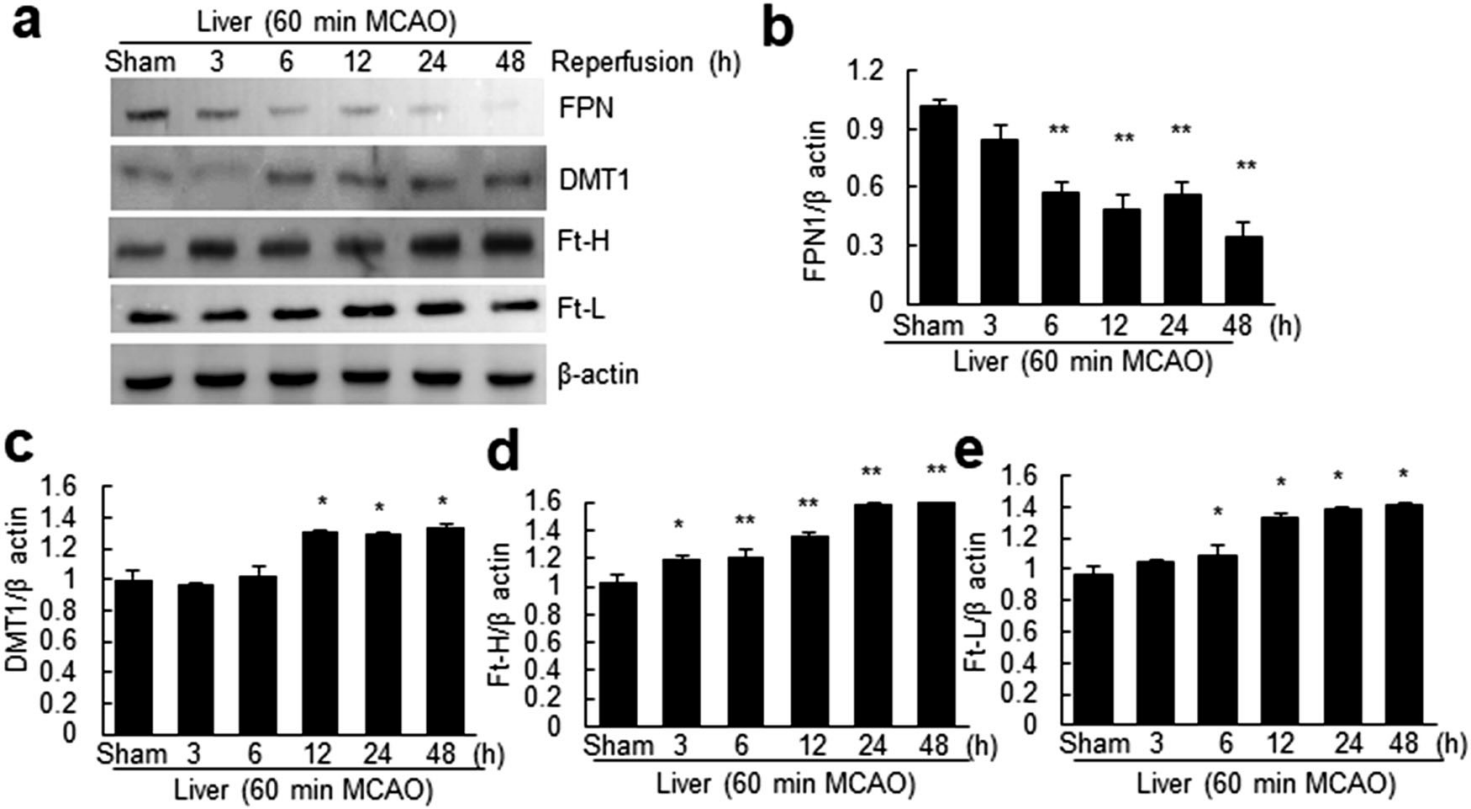
Temporal changes in the expression of iron-related molecules in the liver after MCAO. Protein samples were obtained from the liver at 3, 6, 12, 24, and 48 h post-MCAO, and the levels of FPN, DMT1, Ft-L, and Ft-H were assessed using immunoblotting. Representative images are shown (**a**), and the results are presented as the means ± SEMs (*n* = 3) (**b**–**e**). *p < 0.05 and **p < 0.01 compared with the sham-operated group.

Elevated hepatic iron levels and intracellular iron accumulation in hepatocytes prompted us to examine whether the expression of iron regulatory proteins was altered in the liver after brain ischemic injury. FPN levels decreased significantly at 6 h after MCAO and remained low until 48 h after MCAO (Fig. 3a, b). Conversely, the levels of DMT1 (the iron importer), ferritin heavy chain (Ft-H), and ferritin light chain (Ft-L) were gradually and significantly increased in the liver after MCAO (Fig. 3a, c–e). These findings collectively indicate that the increase in intracellular iron levels in the liver following cerebral ischemic injury is accompanied by the dynamic regulation of several iron regulatory proteins.

### Hepcidin induction in the liver after cerebral ischemic injury

**Fig. 4:**
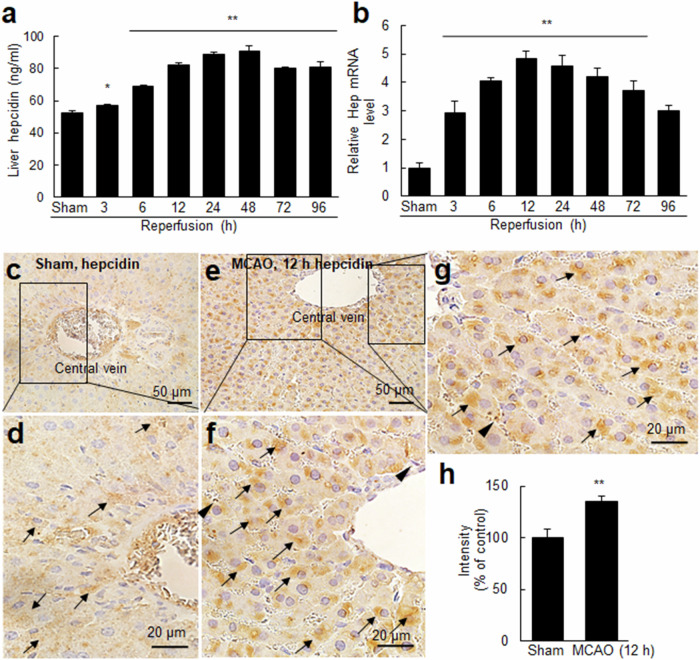
Hepcidin induction in the liver after a cerebral ischemic insult. Hepcidin protein (**a**) and hepcidin mRNA (**b**) levels were determined in liver samples obtained at 3, 6, 12, 24, 48, 72, and 96 h after MCAO using ELISA and RT‒qPCR, respectively, and the results are presented as the means ± SEMs (*n* = 4). *p < 0.05 and **p < 0.01 compared with the sham control group. Liver tissue sections from sham controls (**c**–**d**) and the MCAO group (**e**–**g**) were prepared at 12 h post-MCAO and subjected to immunohistochemistry using an anti-hepcidin antibody, followed by counterstaining using H&E. **h** The staining intensity was measured using ImageJ software, and the quantified results are presented as the means ± SEMs (*n* = 18 from 3 animals). **p < 0.01 compared with the sham control groups Arrows indicate hepcidin immunoreactivity in hepatocytes, and arrowheads indicate hepcidin immunoreactivity in Kupffer cells. The scale bars in **c** and **e** represent 50 µm, and those in **d**, **f**, and **g** represent 20 µm.

We then investigated whether hepcidin expression is also induced in the liver after MCAO. ELISA revealed a significant increase in hepcidin protein levels in the liver parenchyma as early as 3 h after MCAO, and this increase continued until 48 h after MCAO (Fig. 4a). Subsequently, hepcidin levels gradually decreased but remained elevated until 96 h after MCAO (Fig. 4a). RT-qPCR analysis revealed that hepcidin mRNA levels increased significantly at 3 h after MCAO, followed by a more rapid increase, reaching a peak of 5-fold induction at 12 h after MCAO (Fig. 4b). While hepcidin mRNA levels gradually decreased, the upregulation of hepcidin expression persisted until 96 h (Fig. 4b). Immunohistochemistry using an anti-hepcidin antibody revealed hepcidin immunoreactivity in hepatocytes of the sham control group (Fig. 4c, d). At 12 h after MCAO, the number of hepcidin-positive hepatocytes increased, and the staining intensity of these cells was significantly greater than that in the sham control group (Fig. 4e–h, arrows). Hepcidin immunoreactivity was rarely detected in Kupffer cells, and when present, the intensity was very low (Fig. 4f, g, arrowheads; Supplementary Fig. 4). These findings show that hepcidin is predominantly upregulated in hepatocytes following cerebral ischemia.

### HMGB1 induction and nuclear-to-cytoplasmic translocation in hepatocytes after cerebral ischemia

**Fig. 5:**
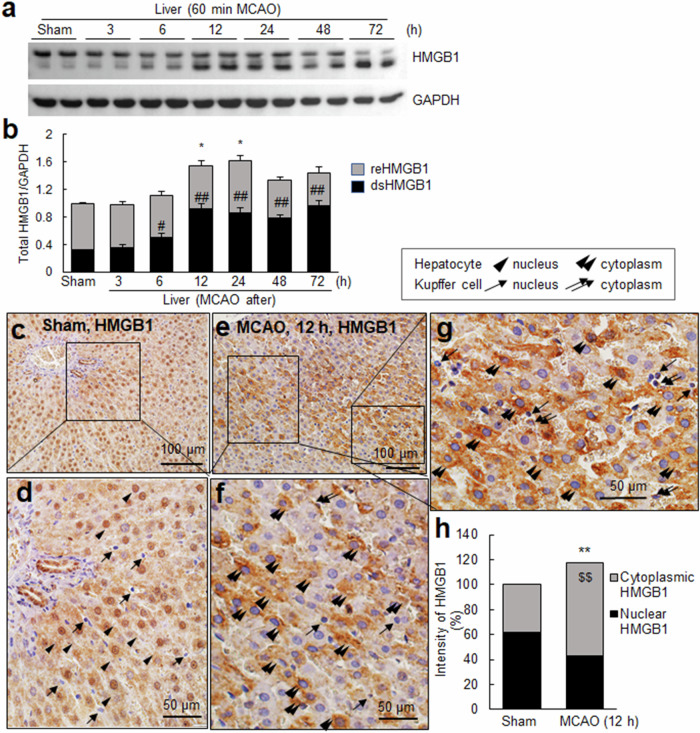
HMGB1 induction in the liver after a cerebral ischemic insult. **a**, **b** HMGB1 levels in the liver parenchyma were determined using immunoblotting at 3, 6, 12, 24, 48, and 72 h after MCAO. Representative blots are shown in **a**, and the quantified results for reHMGB1 and dsHMGB1 levels are presented as the means ± SEMs (*n* = 5) in **b**. Liver tissue sections were prepared from the sham control (**c**, **d**) and MCAO groups (**e**–**g**) at 12 h post-MCAO. Immunohistochemistry was performed using an anti-HMGB1 antibody, followed by counterstaining with H&E. Representative images are shown in **c**–**g**, and the quantified results for nuclear and cytoplasmic hepcidin levels are presented as the means ± SEMs (*n* = 20) in **h**. Arrowheads and double arrowheads indicate HMGB1 immunoreactivity in the nucleus or cytoplasm of hepatocytes, respectively, and arrows and double arrows indicate HMGB1 immunoreactivity in the nucleus or cytoplasm of Kupffer cells, respectively. The scale bars in **c** and **e** represent 100 µm, and those in **d**, **f**, and **g** represent 50 µm. *p < 0.05 and **p < 0.01 compared with the sham-operated group (total amounts), ^#^*p* < 0.05 and ^##^*p* < 0.01 compared with the sham-operated group (dsHMGB1), and $$ *p* < 0.01 compared with the sham-operated group (cytoplasmic HMGB1).

In a previous study, we reported that HMGB1 accumulates significantly in serum as early as 1 h after MCAO, with continuous and rapid increases until 24 h^20^. Considering the increased intracellular iron levels and hepcidin induction in hepatocytes following MCAO, we investigated whether HMGB1 expression is also induced in the liver and involved in hepcidin induction in hepatocytes. The results obtained from the immunoblot analysis revealed that HMGB1 expression was significantly induced 12 h after the ischemic insult, with a further enhancement at 24 h (Fig. 5a, b). In the sham control group, two types of HMGB1, reHMGB1 and dsHMGB1, were detected, with reHMGB1 levels significantly higher than those of dsHMGB1 (Fig. 5a, b). Notably, the major type of HMGB1 induced in the liver after MCAO was dsHMGB1, which was significantly induced as early as 6 h after ischemic insult and further increased at 12 h, and the significant increase in the level of dsHMGB1 persisted until 72 h (Fig. 5a, b). Immunohistochemistry using an anti-HMGB1 antibody revealed that in sham control animals, HMGB1 was detected in the nuclei of almost all liver cells (Fig. 5c, d, arrowheads). Interestingly, at 12 h after MCAO, HMGB1 immunoreactivity was significantly increased in hepatocytes, and a marked alteration in its subcellular localization was detected mainly in the cytoplasm (Fig. 5e–h, double arrowheads). Collectively, these results suggest that hepatocytes are the primary cell type responsible for HMGB1 induction in the liver after MCAO, accompanied by evident HMGB1 translocation from the nucleus to the cytoplasm.

### HMGB1 induction in hepatic Kupffer cells after cerebral ischemia

**Fig. 6:**
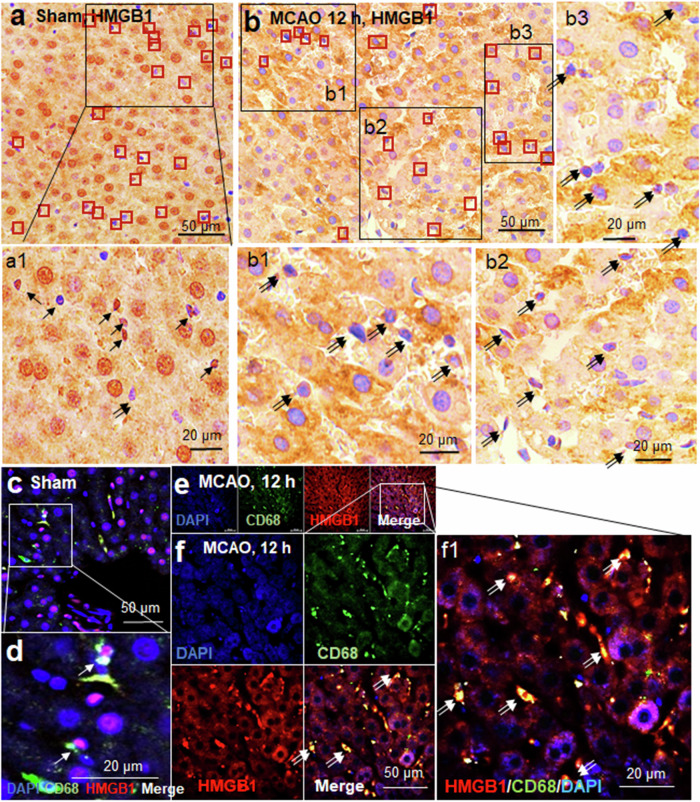
HMGB1 induction in Kupffer cells after a cerebral ischemic insult. Liver tissue sections were prepared from the sham control (**a** and **c**) and MCAO (**b**, **d**) groups at 12 h post-MCAO. **a**, **b** Immunohistochemistry was performed using an anti-HMGB1 antibody, followed by counterstaining with H&E. **c**–**f** Triple fluorescence immunohistochemistry was performed using an anti-CD68 antibody, an anti-HMGB1 antibody, and DAPI. Representative images are shown. Arrows and double arrows indicate HMGB1 immunoreactivity in the nucleus or cytoplasm of Kupffer cells, respectively. The scale bars in **a**–**c** and **f** represent 50 µm, and those in a1, b1-b3, d, and f1 represent 20 µm.

Kupffer cells are much smaller than hepatocytes and reside within the sinusoids of the liver. Immunohistochemistry using an anti-HMGB1 antibody revealed HMGB1 localization in Kupffer cells, primarily in the nuclei of the cells in the sham control group (arrows in Fig. 6a1; Fig. 5d, f, g). In contrast, at 12 h after MCAO, HMGB1 immunoreactivity was detected mainly in the cytoplasm of Kupffer cells (double arrows in Fig. 6b1–b3; Fig. 5f, g). However, the number of HMGB1-positive cells and the HMGB1-stained area of these cells were smaller than those of hepatocytes. Triple fluorescence immunohistochemical staining using anti-HMGB1, anti-CD68 (Kupffer cell marker), and DAPI was performed to further confirm the localization of HMGB1 in Kupffer cells. In sham controls, HMGB1 immunoreactivity was detected in the nuclei of CD68-positive cells (arrows in Fig. 6c, d). However, at 12 h after MCAO, HMGB1 immunoreactivity was detected in CD68-positive cells, primarily in the cytoplasm (double arrows in Figs. 6e, f, f1). Collectively, these findings indicate that HMGB1 expression is induced in Kupffer cells, although the amount of HMGB1 produced by Kupffer cells is likely to be relatively lower than that produced by hepatocytes.

### Disulfide and reduced HMGB1 induce hepcidin upregulation in hepatocytes via TLR4/RAGE- or CXCR4/RAGE-dependent mechanisms, respectively

**Fig. 7:**
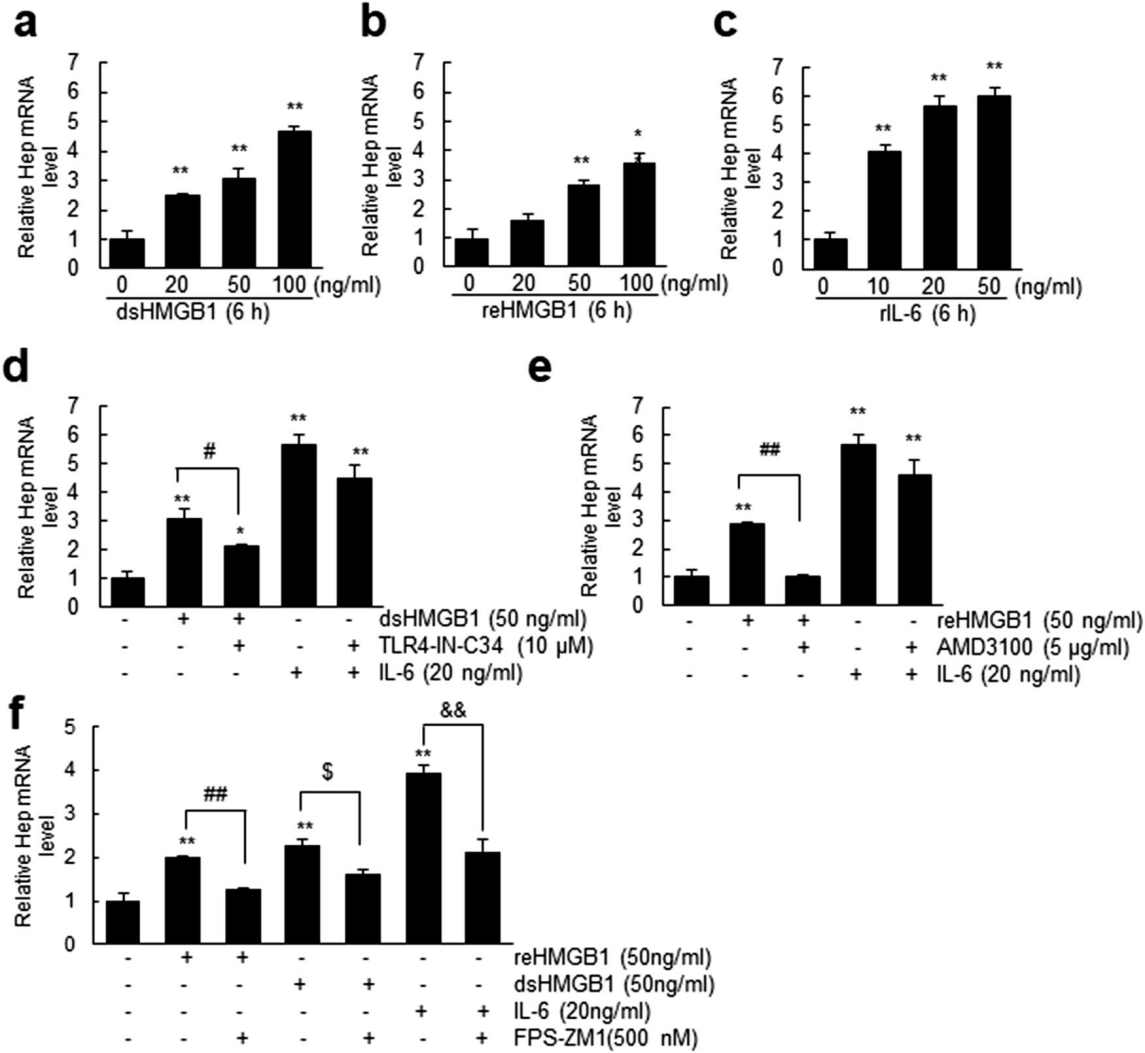
Both dsHMGB1 and reHMGB1 induce hepcidin expression in hepatocytes. Hepcidin expression levels were determined using RT‒qPCR after incubating AML12 cells with dsHMGB1 (20, 50, or 100 ng/mL) (**a**), reHMGB1 (20, 50, or 100 ng/mL) (**b**), or IL-6 (10, 20, or 50 ng/mL) (**c**) for 6 h.AML12 cells were incubated with dsHMGB1 (50 ng/mL) or IL-6 (20 ng/mL) in the presence or absence of TLR4-IN-C34 (10 µM) (**d**) or with reHMGB1 (50 ng/mL) or IL-6 (20 ng/mL) in the presence or absence of AMD3100 (5 µM) (**e**) for 6 h. **f** AML12 cells were incubated with dsHMGB1 (50 ng/mL), reHMGB1 (50 ng/mL), or IL-6 (20 ng/mL) in the presence or absence of FPS-ZM1 (500 nM) for 6 h. Hepcidin mRNA levels were determined using RT‒qPCR. The results are presented as the means ± SEMs (*n* = 4), *p < 0.05 and **p < 0.01 compared with sham controls; ^##^*p* < 0.01, ^$^*p* < 0.05, and ^& &^*p* < 0.01 for comparisons between the indicated groups.

Since dsHMGB1 expression was induced in the liver after MCAO, we investigated its ability to upregulate hepcidin expression in hepatocytes. Treatment of AML12 cells, a hepatocyte cell line, with recombinant dsHMGB1 (20, 50, or 100 ng/mL) for 6 h resulted in a significant increase in hepcidin expression at all doses, including 20 ng/mL, as determined using RT‒qPCR (Fig. 7a). Treatment with reHMGB1 (20, 50, or 100 ng/mL) also upregulated hepcidin expression but to a relatively lesser extent (Fig. 7b). Interestingly, an examination of the hepcidin-inducing capacity of IL-6, a known inducer of hepcidin expression in hepatocytes^27^ that is upregulated in the liver after MCAO (Supplementary Fig. 5), revealed increased levels of hepcidin induction (Fig. 7c). Collectively, these findings suggest that both dsHMGB1 and reHMGB1 can upregulate hepcidin expression in hepatocytes, albeit at a relatively lower level than IL-6. Importantly, dsHMGB1- and reHMGB1-mediated hepcidin induction in AML12 cells was significantly inhibited by cotreatment with TLR4-IN-C34 (10 μM, an inhibitor of TLR4) or AMD3100 (5 µg/ml, a CXCR4 antagonist), respectively (Fig. 7d, e). Interestingly, cotreatment with FPS-ZM1 (500 nM, a RAGE antagonist) significantly inhibited the induction of hepcidin expression by dsHMGB1 or reHMGB1 (Fig. 7f). These results indicate that TLR4, CXCR4, and RAGE are involved in dsHMGB1- and reHMGB1-mediated hepcidin induction in hepatocytes. Notably, IL-6-mediated hepcidin induction in hepatocytes was not inhibited by treatment with TLR4-IN-C34 or AMD3100 but was inhibited by treatment with FPS-ZM1 (Fig. 7d–f). Taken together, these findings suggest that the endogenous TLR4, CXCR4, and RAGE signaling pathways play a role in dsHMGB1- and reHMGB1-mediated hepcidin induction in hepatocytes.

### Both ROS induced during MCAO–reperfusion injury and HMGB1 released after MCAO cause ROS production in the liver and hepatocyte activation

**Fig. 8:**
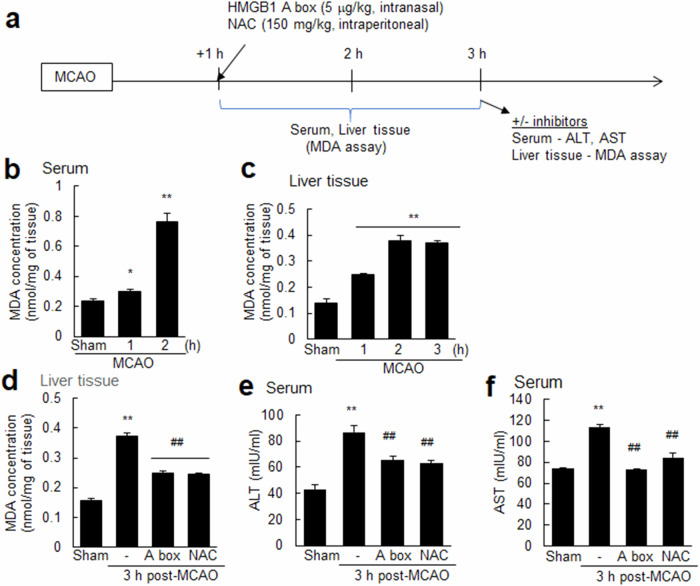
NAC and HMGB1 A box treatments reduce liver ROS production and the subsequent activation of liver cells. **a** NAC (150 mg/kg) or HMGB1 A box (5 µg/kg) was administered intraperitoneally or intranasally, respectively, at 1 h after MCAO. **b**, **c** MDA levels were determined in the serum and liver tissue at 1 and 2 h post-MCAO and at 1, 2, and 3 h post-MCAO, respectively, using assay kits. **d** Liver MDA levels were assessed at 3 h post-MCAO after treatment with or without NAC or HMGB1 A box using assay kits. Serum levels of ALT (**e**) and AST (**f**) were measured at 3 h after MCAO after treatment with or without NAC or HMGB1 A box using ELISAs. The data are presented as the means ± SEMs (*n* = 4). **p < 0.01 compared to sham controls, ##*p* < 0.01 compared to MCAO + saline controls.

Intriguingly, hepatocytes and Kupffer cells in the liver displayed an unexpectedly rapid response to cerebral ischemia, inducing HMGB1 expression and upregulating hepcidin expression. This result prompted us to explore the signaling molecules connecting brain damage to a distant organ, the liver. We measured serum ROS levels using the MDA assay to assess the impact of oxidative stress on the liver following MCAO-reperfusion injury (Fig. 8a). Serum MDA levels exhibited a significant increase starting at 1 h post-MCAO, with a further pronounced increase at 2 h (Fig. 8b). These findings indicate the rapid induction of oxidative stress following MCAO–reperfusion. Interestingly, a significant increase in MDA levels was also detected in the liver tissue as early as 1 h after MCAO, albeit at lower levels than those in the serum (Fig. 8c). This result indicates an early response in the liver following MCAO–reperfusion, initiating local ROS production. We then investigated whether interventions targeting the ROS or HMGB1 produced following MCAO could alter liver ROS levels. N-acetylcysteine (NAC, 150 mg/kg), a ROS scavenger, and HMGB1 A box (HMGB1 antagonistic peptide, 5 mg/kg) were administered intraperitoneally and intranasally, respectively, 1 h after MCAO (Fig. 8a). Both NAC and HMGB1 A box significantly reduced liver ROS levels at 3 h post-MCAO, with comparable levels of suppression observed (Fig. 8d). Importantly, treatment with either NAC or the HMGB1 A box also resulted in significant reductions in the serum ALT and AST levels (Fig. 8e, f). Importantly, the administration of NAC or HMGB1 A box, particularly when administered early, exerts a robust protective effect on the brain. Collectively, these findings suggest that both ROS production induced after MCAO–reperfusion and HMGB1 release after MCAO contribute to liver damage, inducing ROS production in the liver and ultimately leading to hepatocyte activation and liver injury.

## Discussion

In a previous study, we characterized the distinct pattern of serum hepcidin levels following cerebral ischemia, including a delayed onset, delayed peak, and prolonged persistence^20^. The present study revealed that hepcidin upregulation in hepatocytes may contribute to the elevation of serum hepcidin levels and systemic iron regulation after cerebral ischemia, with HMGB1 playing a key role. Elevated serum iron levels and increased serum hepcidin levels have been observed in patients with ischemic^13,24,25^ or hemorrhagic stroke^26^. These findings are significant because they indicate a correlation between serum iron and hepcidin levels^24,25^ and between serum hepcidin levels and patient outcomes^26^. Moreover, due to the disruption of the blood–brain barrier (BBB) following cerebral ischemic injury, iron leakage from the serum into the brain parenchyma can influence the iron content of the brain after ischemia, and vice versa.

Various peripheral organs, including the lung, heart, kidneys, and spleen, respond after cerebral ischemia, either in the context of direct organ damage or as a secondary consequence of brain damage^28–30^. Notably, cerebral ischemia can trigger severe inflammation and subsequent immunosuppression in these organs, which may worsen tissue damage, impede recovery, and increase the risk of infection^28,30^. In the liver, glutamate released from damaged brain tissue reaches the CSF and bloodstream^31^, promoting the synthesis of glutamate–oxaloacetate transaminase (GOT), a liver enzyme that plays a protective role by metabolizing glutamate in the blood^32^. Decreased serum GOT levels and elevated glutamate concentrations have been independently linked to a larger infarct volume and poor functional outcomes^32,33^. Furthermore, the liver is responsible for the synthesis and metabolism of blood coagulation factors and fibrinolytic enzymes, which are essential components of stroke pathophysiology. In the present study, we found that acute and evident histological alterations occur in the liver tissue following cerebral ischemia. This report is, to our knowledge, the first to describe substantial liver tissue damage after cerebral ischemia. Hepatocyte vacuolization was clearly detected throughout the liver tissue along with prominent hepatic cord disruptions and sinusoid dilation. Additionally, a clear accumulation of neutrophils was observed in the liver sinusoid and increased infiltration of these cells into the liver parenchyma. The observation of such rapid and severe injury in a distant peripheral organ such as the liver following cerebral ischemia was unexpected. The rapid increase in the serum ALT and AST levels as early as 3 h post-MCAO suggested that ROS may act as potential signaling molecules, communicating damage from the brain to distant liver cells. Reperfusion injury occurs after a transient cerebral ischemic episode when blood flow and tissue oxygenation are restored^34,35^. Although various mechanisms have been implicated in reperfusion injury, excessive ROS production is considered a key contributor^34,35^. Our study demonstrated that ROS scavenging with NAC administration at 1 h after MCAO significantly reduced both local (liver) ROS production and the increases in serum ALT and AST levels observed at 3 h post-MCAO (Fig. 8d–f). These findings strongly support the hypothesis that ROS generated during reperfusion injury contribute to the activation of liver cells.

Our findings support liver cell activation following cerebral ischemia, as evidenced by the significant and rapid increase in the serum levels of both ALT and AST following cerebral ischemia. This finding aligns with previous research showing a positive correlation between ALT levels and stroke^36,37^. Additionally, we observed a significantly lower AST/ALT ratio after cerebral ischemia (Supplementary Fig. 6), consistent with prior reports suggesting a link between a low AST/ALT ratio and cardiovascular disease, including stroke^38^. While numerous studies have established an association between ischemic stroke and various liver diseases, including nonalcoholic fatty liver disease (NAFLD), liver cirrhosis, and liver fibrosis, along with an increased risk of stroke^39–41^, research on the causal effects of stroke on liver function remains limited. Further investigations are necessary to fully understand the complex responses of the liver to cerebral ischemia.

Given the rapid release of HMGB1 from damaged neurons after stroke and its accumulation in serum as early as 1 h after MCAO^20^, we propose two potential sources of HMGB1 following cerebral ischemia: (1) locally synthesized HMGB1 within the liver, acting in an autocrine or paracrine manner, and (2) transported HMGB1 originating from damaged brain tissue, reaching the liver via the bloodstream and exerting its effect in an endocrine manner. As a paracrine source, activated hepatocytes appear to be the primary producers of local HMGB1, as evidenced by the observed nuclear-to-cytoplasmic translocation of HMGB1 in these cells. However, the contributions of other cell types, such as Kupffer cells, stellate cells, and endothelial cells, cannot be entirely excluded. Kupffer cell-derived HMGB1 has been linked to liver injury, including liver transplantation^42,43^, and damage in remote tissue caused by intestinal ischemia/reperfusion^44^ and sepsis^45^, our current study suggested that the contribution of these cells to HMGB1 levels in cerebral ischemia might be relatively low compared to hepatocytes. Nevertheless, further investigation is warranted to delineate the potential contributions of these cell types to HMGB1 levels in cerebral ischemia. In addition to the local induction of HMGB1 expression in the liver, the substantial increase in serum HMGB1 levels observed after stroke (almost 10-fold)^20^ supports a potential role for transported serum HMGB1 in hepcidin induction by hepatocytes. Furthermore, the significant reductions in local (liver) ROS production and serum ALT and AST levels at 3 h post-MCAO following intranasal HMGB1 A box administration at 1 h after MCAO (Fig. 8d–f) strongly suggest that HMGB1 released from damaged neurons can reach the liver and activate hepatocytes, potentially contributing to hepcidin induction. Similarly, other proinflammatory mediators released during brain ischemia, such as cytokines, necrotic cell fragments, and toxic substances, might play similar roles. In particular, IL-6, a known inducer of hepcidin expression in hepatocytes, could be especially important. Notably, our supplementary data (Supplementary Fig. 5) indicated that IL-6 is not only delivered to the liver via serum but its expression is also induced locally, potentially more potently contributing to hepcidin induction in hepatocytes. Therefore, HMGB1, along with other factors, may be delivered through multiple sources and induce hepcidin expression in the liver.

The critical roles of HMGB1 in various liver pathologies, including liver transplantation^42,43,46,47^ and fibrosis^48^, are well established^49^. Interestingly, our study revealed a conversion of HMGB1 in the liver from its reduced form to its disulfide form following cerebral ischemia. Notably, dsHMGB1 is the most potent proinflammatory form and is known to bind to TLR4^50^. TLR4 signaling has been implicated in hepcidin upregulation in various cell types, including lipopolysaccharide (LPS)-treated hepatocytes^27^ and retinal pigment epithelium^51^, and in an animal model of ICH^52^. Furthermore, the crucial role of dsHMGB1 has also been documented in liver pathologies such as transplantation^42,53^. Further investigation is necessary to elucidate the specific functions of different HMGB1 subtypes in the liver and their potential contributions to various liver diseases.

Our study revealed signaling pathways linking cerebral ischemia with systemic iron dysregulation. Ischemia triggers hepatocyte activation and liver injury, which in turn stimulate the liver to produce HMGB1. This newly synthesized HMGB1, primarily in the disulfide form (dsHMGB1), along with serum HMGB1 derived from the ischemic brain, upregulates hepcidin expression in hepatocytes. Elevated levels of hepcidin, the master regulator of iron homeostasis, restrict iron availability throughout the body. These findings highlight a novel mechanism by which HMGB1 bridges the gap between cerebral ischemia and systemic iron dysregulation. This knowledge provides valuable insights into stroke pathophysiology and suggests potential therapeutic targets for managing the stroke-associated iron imbalance. Furthermore, considering the limited research on distant organ damage, such as liver injury, following cerebral ischemia, the present report establishes a valuable foundation for further investigations.

## Supplementary information

Supplementary materials
