# Supplementary material for: Hepatocyte activation and liver injury following cerebral ischemia promote HMGB1-mediated hepcidin upregulation in hepatocytes and regulation of systemic iron levels
Source: Exp Mol Med. 2024 Oct 1;56(10):2171–83. doi: 10.1038/s12276-024-01314-y (PMC11541749; doi:10.1038/s12276-024-01314-y)
Supplement: Supplementary file 1 — Supplementary materials [file 12276_2024_1314_MOESM1_ESM.pdf]

Supplementary materials

Hepatocyte activation and liver injury following cerebral ischemia promote HMGB1-mediated hepcidin upregulation in hepatocytes and regulation of systemic iron levels

Dashdulam Davaanyam, Song-I Seol, Sang-A Oh, Hahnbi Lee, Ja-Kyeong Lee*

Department of Anatomy, Inha University School of Medicine, Incheon, 22212, Korea

Key words: HMGB1; hepcidin; iron; MCAO; hepatocytes

***Corresponding author:**

Ja-Kyeong Lee, Ph.D.
Department of Anatomy, Inha University School of Medicine
Inharo 100, Inchon, 22202, Republic of Korea
Tel, +82-32-860-9893; jklee@inha.ac.kr

Supplementary figure 1

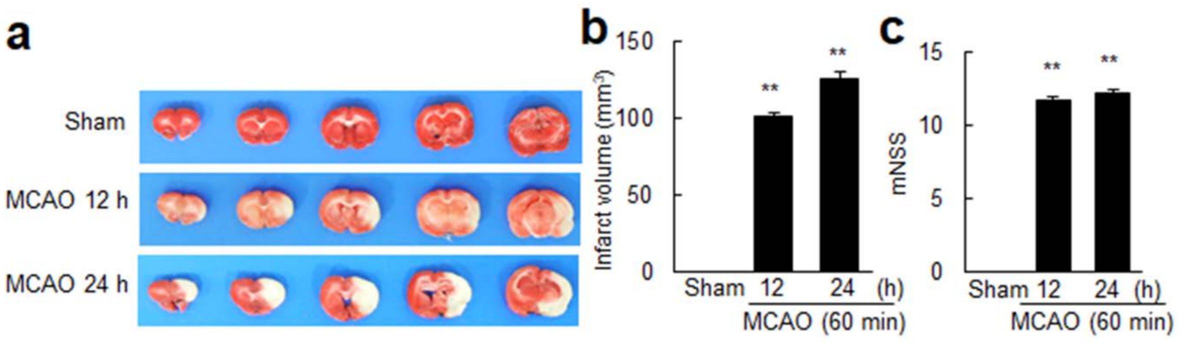

Supplementary figure 1. Infarct volume and neurological deficits of MCAO animals

(a-b) Coronal brain sections were prepared after 12 and 24 h of MCAO, and mean infarct volumes were determined using TTC staining. Representative images of infarctions in coronal brain sections are presented (a), and mean infarction volumes are presented as means ± SEMs (n = 4) (b). (c) Modified neurological severity scores were measured at 12 and 24 h after MCAO and results are presented as means ± SEMs (n = 4). MCAO, saline-treated MCAO group (n = 4); Sham, sham-operated control group (n = 4).

Supplementary figure 2

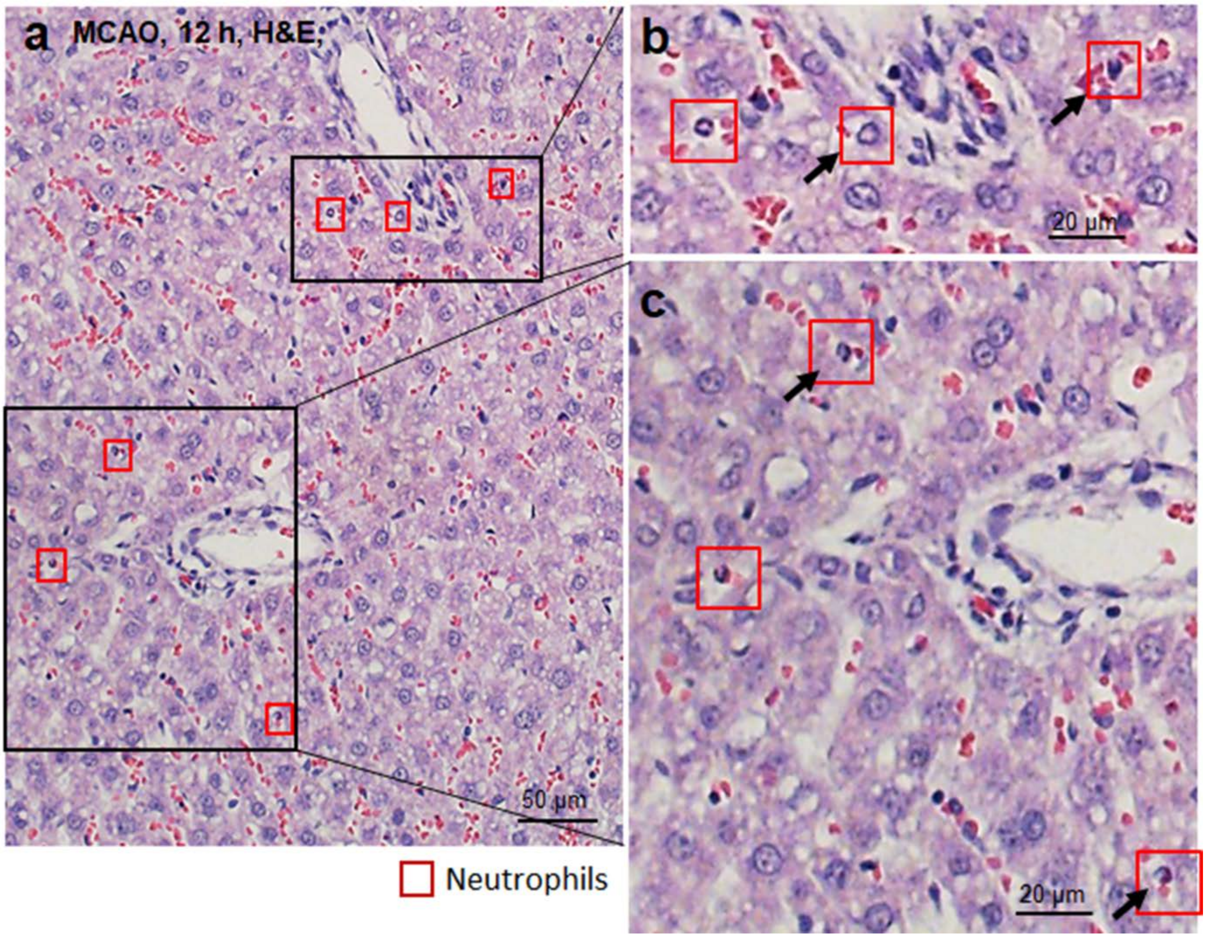

Supplementary figure 2. Neutrophil infiltration in liver parenchyma after cerebral ischemia.

(a) Liver tissue sections were obtained from MCAO groups 12 h after MCAO and stained with H&E. Red boxes indicate neutrophils and arrows indicate infiltrated ones in liver parenchyma. High magnification images were presented in b and c. Scale bars in a represent 50 μm and those in b and c represent 20 μm.

Supplementary figure 3

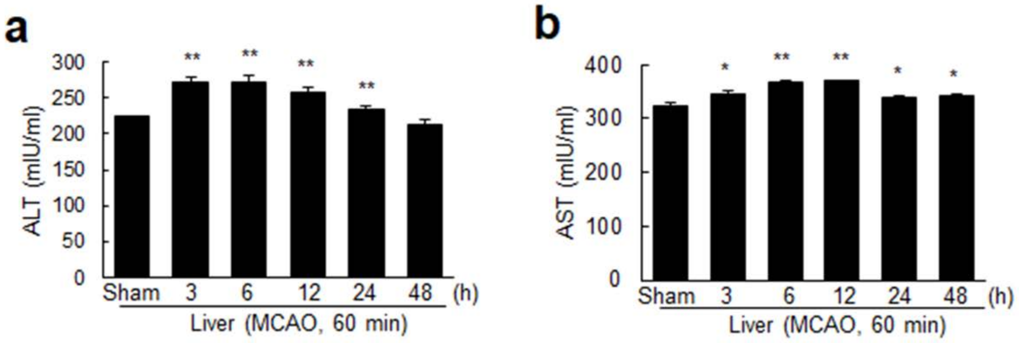

Supplementary figure 3. ALT and AST levels in liver tissue after cerebral ischemic insult

Protein samples were obtained from liver tissue at 3, 6, 12, 24, and 48 h post-MCAO. Levels of ALT (a) and AST (b) were measured using ELISA. Quantified results are presented as means ± SEMs (n=4). $^*p < 0.05$, $^{**}p < 0.01$ vs. Sham controls

Supplementary figure 4

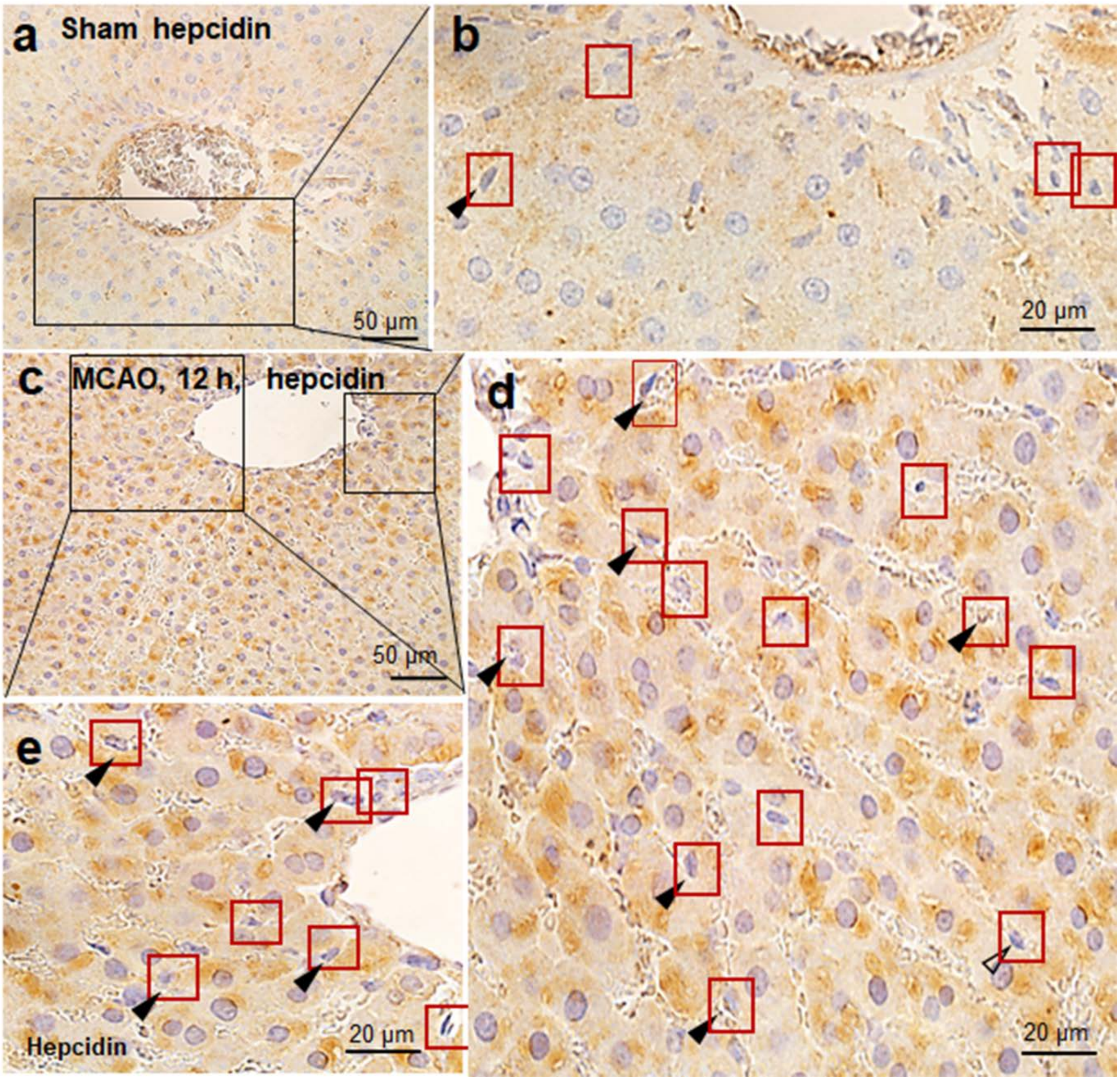

Supplementary figure 4. Hepcidin induction in Kupffer cells after cerebral ischemia

Liver tissue sections were obtained from sham controls (a-b) and MCAO group (c-e) at 12 h after MCAO and subjected to immunohistochemistry using anti-hepcidin antibody, followed by counter staining using H&E. Red boxes indicate Kupffer cells and arrowheads indicate hepcidin immunoreactivity in Kupffer cells. The scale bars in a and c represent 50 μm and those in b, d, and e represent 20 μm.

Supplementary figure 5

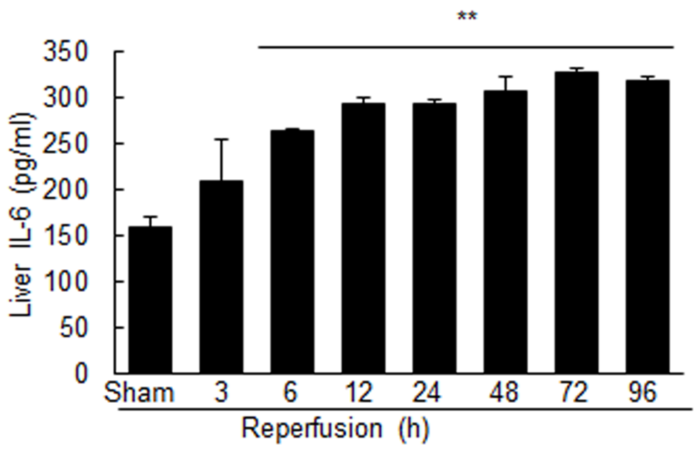

Supplementary figure 5. IL-6 induction in liver after cerebral ischemic insult

Levels of IL-6 in liver at 3, 6, 12, 24, 48, 72, and 96 h after MCAO were assessed using ELISA, and results are presented as means ± SEMs (n = 3). ** $p < 0.01$ vs. Sham-operated groups.

Supplementary figure 6

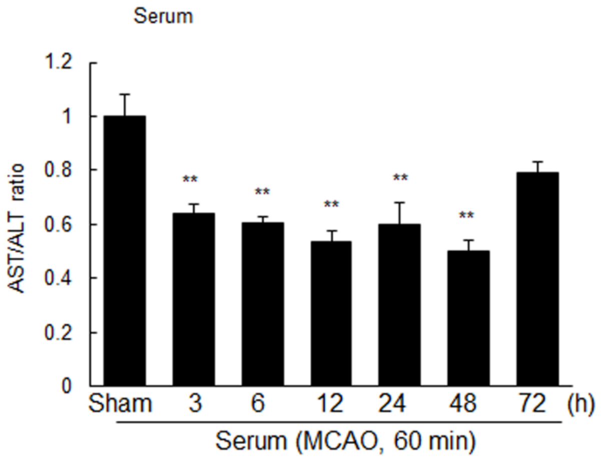

Supplementary figure 6. Serum AST/ALT ratio following cerebral ischemia

Serum levels of ALT and AST were measured at 3, 6, 12, 24, 48, and 72 h after MCAO using ELISA (Fig. 1h and i), and AST/ALT ratio are presented as means ± SEMs (n = 4). **$p < 0.01$ vs. Sham controls.
